# Supplementary material for: RIPK1-Induced A1 Reactive Astrocytes in Brain in MPTP-Treated Murine Model of Parkinson’s Disease
Source: Brain Sci. 2023 Apr 27;13(5):733. doi: 10.3390/brainsci13050733 (PMC10216483; doi:10.3390/brainsci13050733)
Supplement: Supplementary file 1 [file brainsci-13-00733-s001.zip › brainsci-2331525-supplementary.pdf]

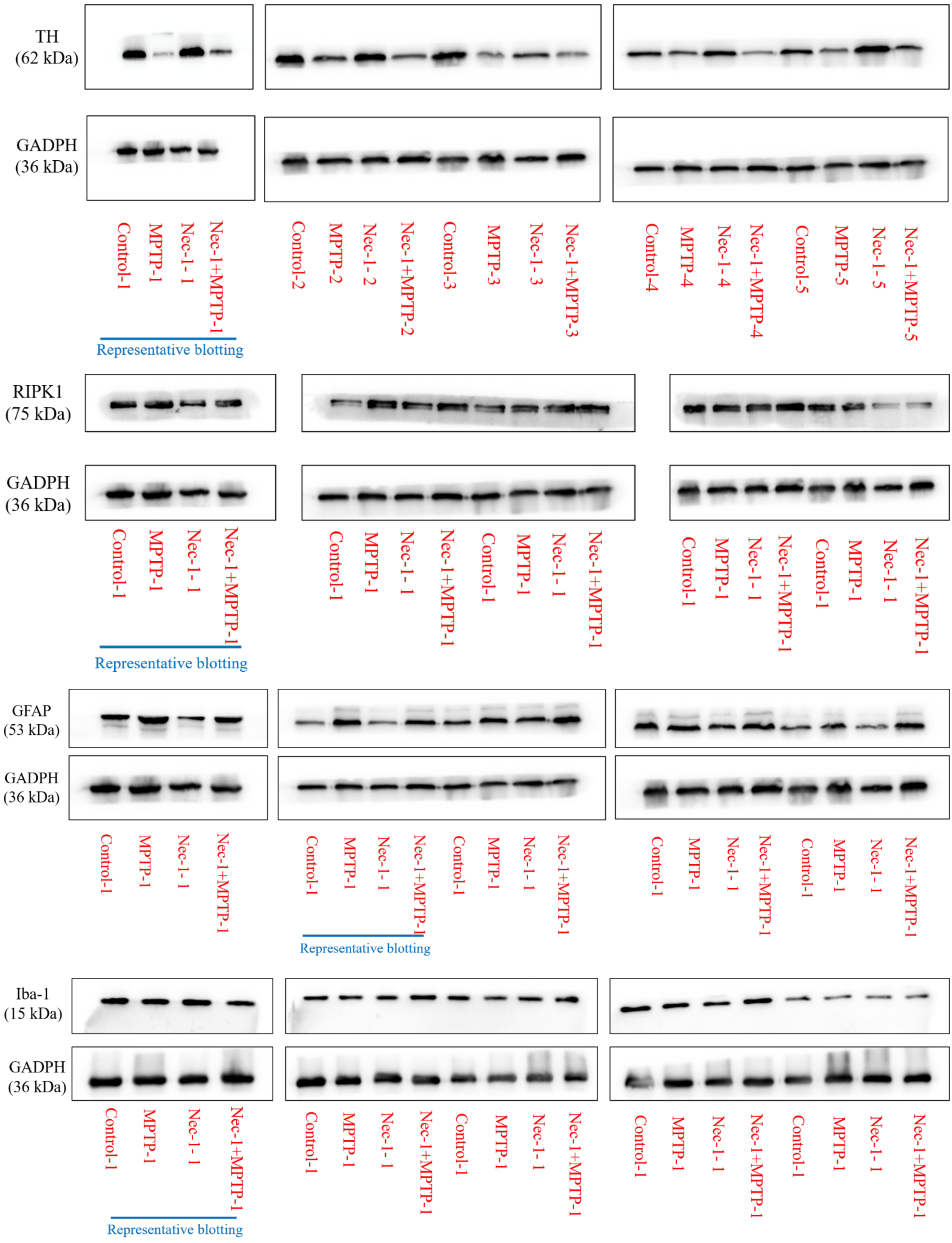

Figure S1: All original Western blot images.

We cut out the target protein bands of TH (62kDa), RIPK1 (75kDa), GFAP (53kDa), and Iba-1 (15kDa) based on their molecular weights during the incubation of an antibody and immerse the bands completely in separate antibody working solutions for incubation.
